# Laser-Assisted Reduction of Highly Conductive Circuits Based on Copper Nitrate for Flexible Printed Sensors

**DOI:** 10.1007/s40820-017-0139-3

**Published:** 2017-03-21

**Authors:** Shi Bai, Shigang Zhang, Weiping Zhou, Delong Ma, Ying Ma, Pooran Joshi, Anming Hu

**Affiliations:** 1 Institute of Laser Engineering, Beijing University of Technology, 100 Pingle Yuan, Beijing, 100124 People’s Republic of China; 2 School of Civil and Transportation Engineering, Beijing University of Civil Engineering and Architecture, Zhanlanguan Road, Beijing, 100044 People’s Republic of China; 3 Oak Ridge National Laboratory, Oak Ridge, TN 37831-6061 USA; 4 Department of Mechanical Aerospace and Biomedical Engineering, University of Tennessee Knoxville, 1512 Middle Drive, Knoxville, TN 37996 USA

**Keywords:** Laser direct writing, Copper circuit, Stretchable sensor, Laser reduction

## Abstract

Stretchable electronic sensing devices are defining the path toward wearable electronics. High-performance flexible strain sensors attached on clothing or human skin are required for potential applications in the entertainment, health monitoring, and medical care sectors. In this work, conducting copper electrodes were fabricated on polydimethylsiloxane as sensitive stretchable microsensors by integrating laser direct writing and transfer printing approaches. The copper electrode was reduced from copper salt using laser writing rather than the general approach of printing with pre-synthesized copper or copper oxide nanoparticles. An electrical resistivity of 96 μΩ cm was achieved on 40-μm-thick Cu electrodes on flexible substrates. The motion sensing functionality successfully demonstrated a high sensitivity and mechanical robustness. This in situ fabrication method leads to a path toward electronic devices on flexible substrates.

**Electronic supplementary material:**

The online version of this article (doi:10.1007/s40820-017-0139-3) contains supplementary material, which is available to authorized users.

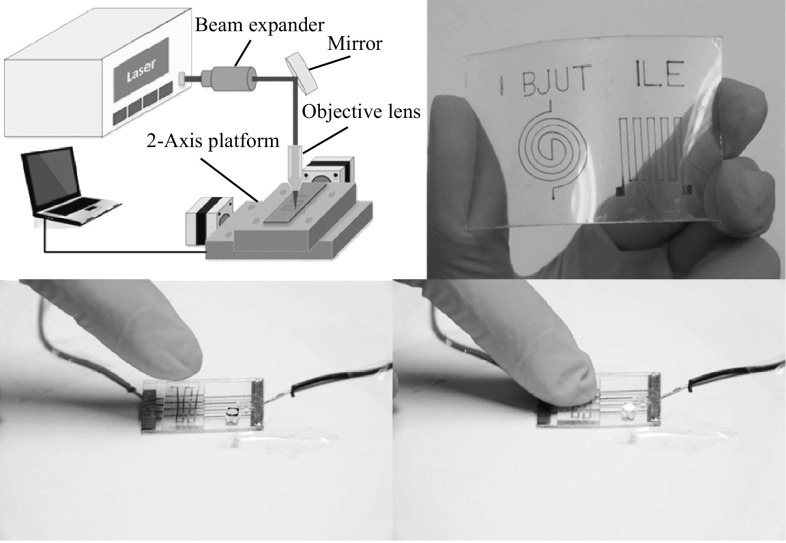

## Highlights

- A novel method to fabricate flexible sensors based on conductive copper circuits by laser-assisted reduction is introduced.
- The copper nitrate hydroxide (Cu(OH)(NO_3_)) was reduced by ethylene glycol using laser scanning on glass and polyethylene terephthalate. By transferring the copper electrode onto the polydimethylsiloxane, which acted as stretchable substrate, the electrode exhibits high sensitivity, and its resistivity is as low as ~90 μΩ cm.
- This type of device can be used for motion detection and touch sensors to control electrical devices.

## Introduction

Stretchable sensors based on conductive materials have become an important aspect of public interest. Furthermore, because of their elasticity and mechanical robustness, stretchable sensors have been applied to robotic control [1], entertainment [2, 3], health monitoring [4, 5], and medical care [6]. By printing silver nanoparticle ink on flexible substrates such as polydimethylsiloxane (PDMS) or polyimide (PI), conducting electrodes can be used as strain sensors for human motion detection [1, 2, 7, 8]. For example, attaching the sensors on fingers or arms, human gestures are detected and identified [9, 10].

Gold and silver nanomaterials (nanoparticle or nanowire), carbon blacks and graphene have been explored as conductive materials owing to their high conductivity and chemical stabilities [1, 2, 11–13]. However, the high cost of silver and gold nanomaterial inks is not in line with the goals of the low-cost flexible and printed electronics. The conductivities of emerging carbon blacks and graphene inks are still significantly lower than that of metals [7, 12]. It is therefore essential to develop alternative materials for printable sensors and devices. Both copper and silver have very similar electrical conductivities. However, copper is significantly less expensive. Conducting electrodes based on sintered copper nanoparticles are the focus of various R&D efforts for the development of next-generation flexible devices on paper [14, 15], antenna [16], epidermal electronics [17], and low-temperature packaging [18]. However, the use of pre-synthesized copper nanoparticles or copper oxide nanoparticles increases the cost and complications of processing [18–20]. The development of an in situ process for Cu nanoparticle synthesis is essential to meet the cost, performance, and reliability requirements of wearable electronic applications.

Various methods, including inkjet printing, screen printing, and intense pulsed light irradiation, have been reported for the fabrication of conductive films and/or electrodes by sintering the copper nanoparticles or reducing copper oxide nanoparticles (Table 1) [21–23]. A major challenge with copper nanoparticle printing is the surface oxidation under ambient conditions, which significantly limits the conductivity. Therefore, the copper ion source has to be reduced, and the reduced copper nanoparticles have to be sintered at the same time and further passivated by capping materials [24].

**Table 1:** Selected examples of fabrication methods for conductive copper electrode

Material	Method	Resistivity (μΩ cm)	References
Cu nanoparticle	Ink jet	20	[19]
Cu nanowire	Ink jet	–	[21]
CuO	Laser reduction	31	[20]
Cu(OH)(NO_3_)/Cu(NO_3_)_2_	Laser reduction	240	[29]
Cu(OH)PO_4_	Laser direct structuring	9.18	[30]
Cu(OH)_2_	Intense pulsed light	5.27	[31]
Cu(OH)(NO_3_)	Intense pulsed light	125.1	[25]
CuO	Intense pulsed light	10	[32]
Cu particle	Flash light sintering	80	[33]

Herein, we report a novel method that directly fabricates copper electrodes from copper salt, rather than pre-synthesized nanoparticle ink. Without using copper oxide as the copper source, the residual non-conducting oxide, which exists at the inner parts of reduced copper pattern, can be avoided [20, 25]. Laser reduction is also convenient to pattern the electrodes with tailored physical and chemical properties [26–28]. Moreover, compared with two-dimensional plane processing with an intense pulsed light method, the advantages of laser direct writing also stem from the fact that laser direct writing is a cost-effective and facile three-dimensional (3D) precise fabrication method, which offers direct writing capability. Consequently, this leads to a significant reduction in the number processing steps, and the material wastage associated with the traditional techniques, such as vacuum deposition and photolithography.

Recently, we reported that the copper nitrate can be directly reduced using a continuous wave laser, on glass [29]. However, the laser reduction mechanism of copper nitrate is still unclear. In this paper, we present detailed investigations on the laser reduction of copper that conducted copper salt, and thereafter elucidated the reduction mechanism with the finite element method (FEM). Our results demonstrated that the reduced copper particles were sintered immediately after laser reduction. After transferring the conducting copper electrodes onto PDMS, a stretchable sensor was developed for electronic devices, such as motion detectors and LED switches.

## Experimental Methods

### Preparation of Cu Salt Ink

To prepare Cu salt ink, 2 mL of ethylene glycol (Tianjin Fu Chen) and 0.5 mL of deionized water were poured into a beaker, and 2.5 g of copper (II) nitrate trihydrate (Cu(NO_3_)_2_·3H_2_O) (Tianjin Fu Chen) was added. The solution was stirred for at least 10 min, to thoroughly dissolve the Cu(NO_3_)_2_. The liquid was heated to 120 °C for 10 min; then the solution took on a black green color and released a large amount of faint yellow gas. After it was cooled down to room temperature (RT), 10 μL of formic acid (Tianjin Fu Chen) was added to the liquid, and the solution was homogenized by ultrasonication, for 5 min. It has been demonstrated that the combination with a carboxyl group (COOH) can improve the solubility of the copper compound in ethylene glycol, which influences the uniformity of the coated copper salt film on the substrate in the next step [31].

### Coating Method and Laser Reduction

**Fig. 1:**
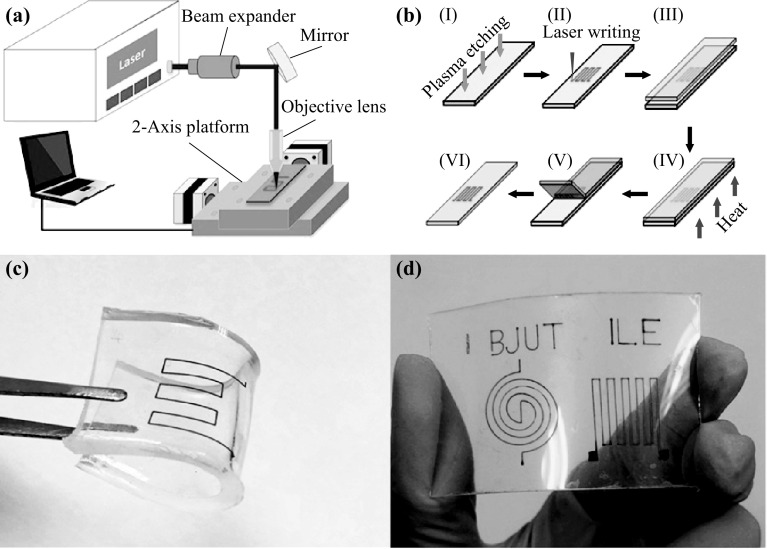
**a** Schematic of the experimental setup for laser direct writing. The laser beam is directed at a collimator. Afterward the beam is reflected by a mirror and focused by a microscope objective (NA = 0.3). The translation stage enables the accurate and repeatable positioning of the substrate in two dimensions, with an overall accuracy of about 500 nm. **b** Schematic illustration of the fabrication of the flexible sensor based on copper electrode: (I) oxygen plasma etching of the surface of the glass, (II) laser writing of copper salt, (III) coating of PDMS onto a reduced copper electrode, (IV) curing of the PDMS-covered substrate, (V) peeling off the PDMS strip from the substrate, (VI) overlay of the PDMS on a target substrate. **c** Photograph of copper electrode on PDMS substrate. **d** Spider and zigzag electrodes on PET substrate

Before coating, the substrate (glass or plastic) was treated by oxygen plasma, to enhance surface adhesion by creating hydrophilic groups. The surface of the substrate was treated by reactive ion etching (RIE) at an applied power of 200 W, for 1 min, in a flowing 0.2 L min^−1^ O_2_ atmosphere. The copper salt layer was deposited using the spin-coating technique, and the spinning rate was maintained at 700 rpm for 10 s, for this study. To write the copper electrodes, a continuous wave laser was used. The employed laser was a diode laser (K808DAECN-30.00 W, BWT Beijing Ltd). The laser beam spot with a diameter of approximately 150 μm (1/e^2^) and 3 nm spectral width (FWHM) was achieved using a microscope objective (NA = 0.3) with a 16-mm work distance. The Beam expander was purchased from Daheng Optics (GCX-L010-SMA-40ac, DHC, Beijing, China). The scanning speed was 5 mm s^−1^. Figure 1a shows the configuration of the laser direct reduction system. After laser processing, the specimen was cleaned using deionized water, in order to wipe off the residual copper salt.

### Strain Sensors Based on Copper Electrode

The first step was to mix a PDMS pre-polymer and a curing agent (Sylgard 184, Dow corning, USA), with a weight ratio of 10:1. After all the bubbles were removed, the mixed liquid was poured onto the glass with the electrode pattern. After curing in a drying oven at 80 °C for 4 h, the PDMS membrane with a thickness of 3 mm was detached from the glass, as shown in Fig. 1b. Finally, the PDMS stripe with the embedded electrode was cut into a size of 5 × 2.5 cm^2^ as a strain sensor (Fig. 1c). Figure 1d shows the typical spider and zigzag electrodes on the polyethylene terephthalate (PET) substrates, obtained by the optimized laser conditions.

### Physical and Chemical Characterization

The high-resolution images of the Cu patterns were captured and measured using field emission scanning electron microscopy (FESEM, Hitachi S-4800, Japan) and transmission electron microscopy (TEM, JEM-2010, JEOL, Japan). The thickness of the Cu pattern was measured by a 3D profiler (Wyko NT1100, Veeco Instruments Inc., USA). The oxidation of the Cu pattern was analyzed using X-ray diffraction at RT (D8 Advance, German). The electrical properties were monitored in real time using an electrochemical workstation (Chenhua CHI600E, China). The Raman spectra were acquired using a confocal microprobe Raman system (in Via-Reflex, Renishaw, UK) with an excitation laser wavelength of 532 nm, power of 2 mW, and the acquisition time was 10 s with two accumulations. Thermal analyses (TGA) were conducted in a thermogravimetric analyzer (Mettler SF/1382 Switzerland). The bending and strain performance of the sensor was evaluated using a tensile tester (Songdun WDW-1, China) and a digital multimeter (Keysight 34475A, USA). To monitor the motion of the finger, the senor was mounted on the finger using strong glue, and the two terminals of the sensor were connected to the lead by conductive silver ink, to reduce the contact resistance between the sensor and copper lead wires. The absorption spectra were measured using a UV–Vis spectrophotometer (UV-9000S, Metash, China).

## Results and Discussion

**Fig. 2:**
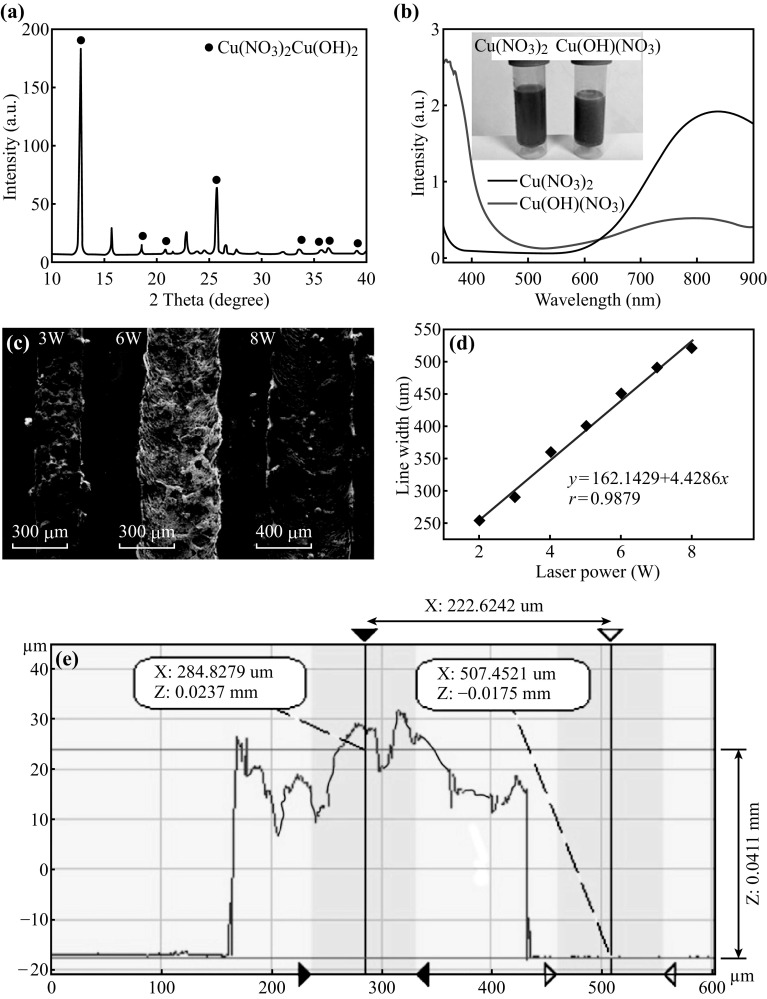
**a** XRD pattern and **b** absorption spectroscopy of copper salt ink (*inset* photographs of copper salts). **c** SEM images of copper line on glass reduced by the laser at powers of 3, 6, and 8 W. **d** Influence of laser power with width of reduced copper line. **e** The height and width of the copper line measured by a 3D profiler

To analyze the main ingredients of the copper salt after heating, we performed X-ray powder diffraction (XRD) and measured the absorption spectrum. As described in the experimental section, when the Cu(NO_3_)_2_ solution is heated, it takes on a black green color, as shown in the inset in Fig. 2b. Figure 2a shows the XRD pattern of the copper salt prepared after heating at 120 °C for 10 min. The peaks at 12.8°, 18.6°, 20.8°, 25.7°, 33.5°, 35.7°, 36.4°, and 39.0° are identified as the peaks of Cu(OH)(NO_3_), having a green color at high concentrations [34]. Other peaks (15.7°, 22.8° and 26.6°) in XRD patterns are assigned to diffraction from copper nitrate trihydrate. In addition, Fig. 2b shows the absorption spectra of Cu(NO_3_)_2_ and Cu(OH)(NO_3_). Both have a peak in the near infrared region at around 800 nm. However, there is a blueshift from 837 to 801 nm, as Cu(NO_3_)_2_ changes to Cu(OH)(NO_3_). Moreover, it also demonstrates that the laser energy will be strongly absorbed when irradiating the specimen using the 808 nm laser. This increase in optical absorbance is favorable for the laser process, which relies on the effective absorbance of the 808 nm laser for decomposition and sintering.

To investigate the impact of oxygen plasma treatment on the substrate, the contact angle was measured. As shown in Fig. S1a and b, the substrate treated with oxygen plasma has lower contact angle values than that of the non-treated substrate. In addition, Figure S1c–g shows that as the copper salt content is increased, the contact angle becomes larger, increasing from 40.8 to 82.7 (0.75–1.75 g mL^−1^) on the PET substrate. Furthermore, because the contact angle influences the thickness of the coated copper salt film, it significantly determines whether or not the bottom layer of the copper ions can absorb enough energy to be reduced by laser. According to our experimental results, the 1.25 g mL^−1^ copper nitrate solution is the optimal condition for coating the film on the substrate. Figure S2 shows a typical SEM image of copper salt. Various shapes can be observed in copper, such as the irregular sphere, rod. The mean size of these particles is about 10 µm.

Figure 2c shows SEM images of the copper line width versus laser power. Briefly, the line width forms a linear relationship (Fig. 2d, the correlation coefficient is: *r* = 0.9879) with the laser power. Experimentally, the reduction cannot happen at a laser power that is lower than 2 W. The reduction threshold is about 2 W, and the line width was approximately 200 μm. However, the notch can be observed on both sides of the copper line (Fig. S3). When the laser power increases to 3 W, the copper line is smooth and results in a better conductivity than that achieved at a lower power. In addition, if the laser power is increased to 6 W or higher, the laser begins to ablate the center of the copper line. The ablated contour is shown in Fig. 2c. Moreover, due to the joining, the laser power can cause the coarsening of copper nanoparticles [35]. Figure S4 shows that the mean size of the nanoparticles changes from 110 to 200 nm, as the laser power is increased. The thickness of the copper line is measured and shown in Fig. 2e. The thickness is about 40 μm higher than the copper line, measured based on CuO ink, which was fabricated by Kang et al. [20] (the thickness of the copper line is about 10 μm). In addition, it can be understood that micropores on the Cu pattern, as shown in Fig. 2e, arise from the gas released from the copper salt [31].

**Fig. 3:**
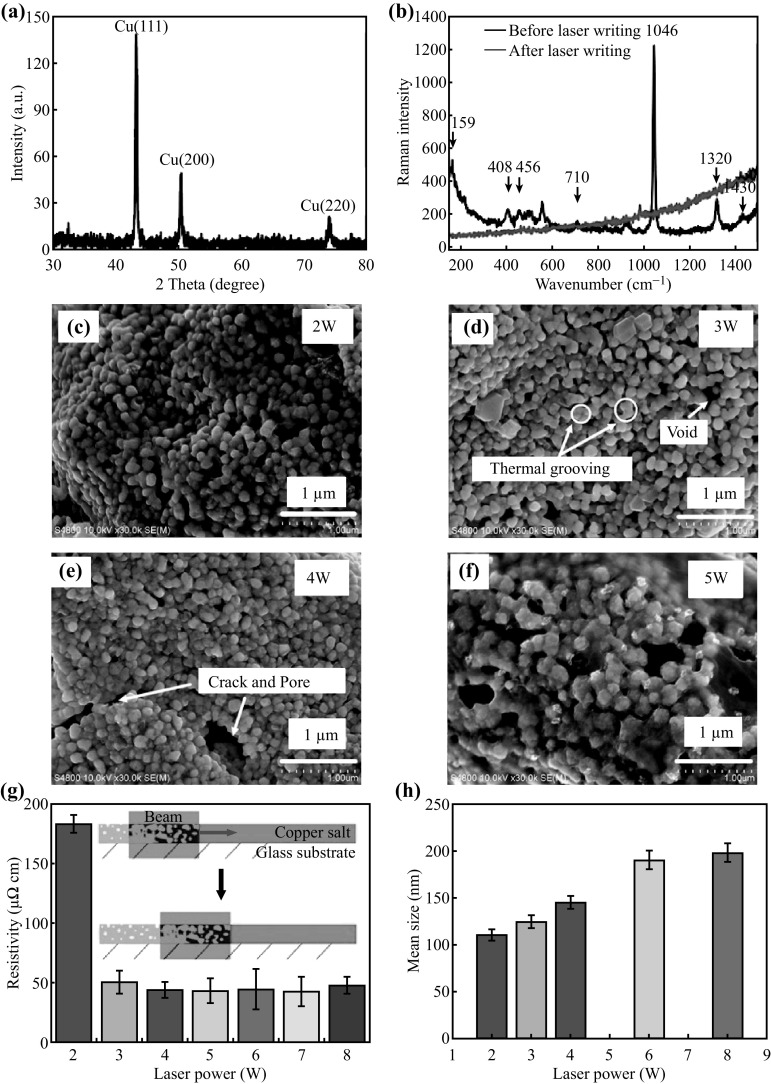
**a** XRD patterns of the copper salt after laser reduction at the laser power of 3 W. **b** Raman spectra of sample before and after laser wring. **c–f** Typical SEM image of copper electrodes reduced by the laser at powers of 2, 3, 4, and 5 W. **g** Resistivity of copper electrode as a function of laser power (*inset* diagram of laser reduction and sintering.). **h** Mean size of copper nanoparticle by laser reduction

Figure 3a shows the XRD peaks of the copper electrode after laser reduction. The diffraction peaks at 43.3°, 50.4°, and 74.2° correspond to copper (JCPDS No. 851326) [36, 37]. The samples visually changed from a black green to bright pink color, as an indicator of the transformation to Cu. As shown in the XRD patterns (Fig. 3a), it is interesting that there are no copper oxide peaks. The observed XRD result clearly indicates that the copper salt is thoroughly reduced and that the copper nanoparticles are not re-oxidized. To verify this analysis, the Raman spectra of the sample are illustrated in Fig. 3b. For the sample before laser writing, the main peaks are found at 1430, 1320, 1046, 710, 456, 408, and 159 cm^−1^. These Raman peaks demonstrate that the copper nitrate is converted into copper nitrate hydroxide after heating. Moreover, the peaks at 1430, 1320, and 1045 cm^−1^ can be assigned to the vibrational modes of the nitrate ions. The bands at 1430 and 1320 cm^−1^ correspond to the symmetric and asymmetric stretching modes of NO_3_^−^, and the band at 1045 cm^−1^ corresponds to the N–O stretching vibration of a mono-dentate O–NO group. The peaks at 456 and 159 cm^−1^ are assigned to a Cu–O vibrational mode and Cu–O–N in-plane bending, respectively [38–40]. After the laser writing, there are no Raman peaks of copper oxide and cuprous oxide. No copper oxide in a reduced copper line is different from the situation with copper oxide as the copper source [20, 22]. The reason for this interesting phenomenon is discussed in the latter parts of this paper. The reduction reactions can be presented as follows [20]:

1 $$2{\text{HO}}({\text{CH}}_{2} )_{2} {\text{OH}} \mathop{\longrightarrow}\limits^{- 2{\text{H}}_{2} {\text{O}}}2{\text{C}}_{2} {\text{H}}_{4} {\text{O}}$$

2 $$\begin{aligned} \xrightarrow{{{\text{Cu}}({\text{OH}})({\text{NO}}_{3} )}}{\text{C}}_{4} {\text{H}}_{6} {\text{O}}_{2} + 2{\text{H}}^{ + } + 2{\text{e}}^{ - } + {\text{Cu}}^{2 + } + {\text{OH}}^{ - } + {\text{NO}}_{3}^{ - } \hfill \\ \to {\text{C}}_{4} {\text{H}}_{6} {\text{O}}_{2} + {\text{Cu}} + {\text{H}}_{2} {\text{O}} + {\text{HNO}}_{3} \hfill \\ \mathop{\longrightarrow}\limits{\text{C}}_{4} {\text{H}}_{6} {\text{O}}_{2} + {\text{Cu}} + {\text{H}}_{2} {\text{O}} + \frac{1}{2}{\text{H}}_{2} {\text{O}} + {\text{NO}}_{2} \uparrow + \frac{1}{4}{\text{O}}_{2} \uparrow \hfill \\ \end{aligned}$$

As the temperature of the solution is increased to the level of about 160–200 °C, the ethylene glycol begins to dehydrate, and leads to the formation of acetaldehyde, which can reduce the copper salt [41]. In the presence of aldehyde, the Cu(OH)(NO_3_) can be directly converted into the elemental form of Cu, as shown in Eq. 2 [25]. Figure 3d, e, f, g shows the morphologies of copper nanoparticles sintered with various laser power levels at a fixed scanning speed. At an initial power of 2 W, the energy applied to the copper salt is low. Although the copper ions are reduced to Cu particles, these particles are isolated and not sintered, as shown in Fig. 3d. Owing to these insulated nanoparticles, the measured resistivity is maintained at ~200 μΩ cm. However, with the assistance of the surface tension and single grain boundary tension [42], the thermal grooving (Fig. 3d) is generated by heating at 3 W. The voids at the grain junctions are observed due to thermal grooving. The resistivity rapidly decreases to 50 μΩ cm. Meanwhile, the micropores and cracks are formed on the Cu patterns at 4 W, due to the bubbling caused by high energy. When the laser power further increases to 5 W, the Cu pattern begins to be destroyed by the evaporation of central parts of pattern. As shown in Fig. 3h, the mean size of the Cu particles increases from 115 to 203 nm, because of laser sintering [35]. It indicates that the size of the copper nanoparticles can be controlled by adjusting the laser power.

In summary, the laser first reduces the cupric ions to elemental Cu, and agglomerated Cu clusters into small nanoparticles. These copper nanoparticles are further sintered by a higher laser power. The sintered copper line can be lifted using tweezers, displaying good sintering mechanical properties (Fig. S8a). Figure S5a shows the typical TEM images of the reduced copper nanoparticles at the laser power of 3 W. The high-resolution TEM image (Fig. S5b) shows the lattice fringes of copper nanoparticles with a *d* spacing of 0.21 nm, corresponding to the (111) plane of the face-centered cubic (FCC) copper. The SAED pattern (Fig. S5c) shows bright rings with a lattice fringe spacing that agrees with the FCC phase of copper [43, 44].

**Fig. 4:**
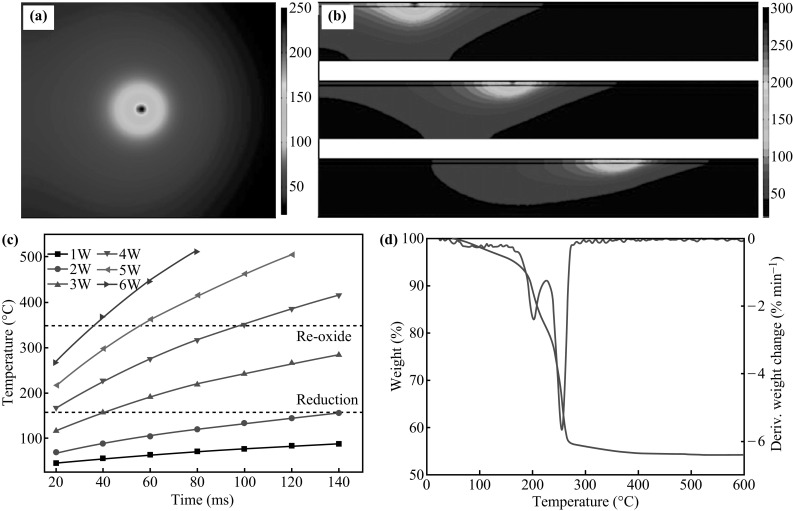
Finite element simulation of laser annealing Cu film. **a** Temperature (degree centigrade) at *k* = 400 W (mK)^−1^, and scanning speed at 5 mm s^−1^ with 3 W inputted power. These conditions resemble the experimental condition. **b** The simulation of temperature profiles at laser scanning speeds of 1, 10, and 20 mm s^−1^. **c** Temperature changing of samples under different laser powers and irradiation times. **d** Thermogravimetric analysis curve of copper nitrate hydroxide

In order to estimate the effect of laser power and irradiation time on the copper salt reduction, we performed the simulation using the finite element modeling. Figure 4a, b, c displays the finite element modeling of laser-annealed Cu films. The conjugate heat transfer module with isothermal surroundings (*T* = 300 K) was used in the simulation. The heat source is fixed as a Gaussian heat source at the surface, with radius = 75 μm. The model is surrounded by air, and the underlying substrate is a typical glass with a thermal conductivity (*k*) of 1.4 W (m K)^−1^. Further simulation details are shown in Fig. S6.

Figure 4a shows the computing temperature profiles at the center of the focused laser spot, using FEM. The profile illustrates that the laser-induced temperature can be increased to 250 °C at a 5 mm s^−1^ scanning speed, with a power of 3 W. This temperature is lower than the 350 °C, at which Cu is oxidized [20, 45]. It has been demonstrated that the thermal decomposition temperature of Cu(OH)(NO_3_) is higher than 238 °C [25, 46]. At such a temperature, the Cu (II) would be directly reduced to Cu (0) by aldehyde. Moreover, HNO_3_ converts to NO_2_, H_2_O, and O_2_ at temperatures higher than 250 °C [25, 47]. However, Kang et al. reported that CuO nanoparticles, being used as a copper source, cannot be completely reduced by the continuous wave laser. This is because the CuO nanoparticles in the central hollow region are not influenced by laser reduction [20]. Figure 4b shows the temperature changes as a function of laser power and irradiation time. As discussed previously, the threshold temperature for laser reduction is about 160 °C, and the re-oxidation temperature of copper particles is about 350 °C. At 3 W, it is clear that the local temperature will be less than 350 °C for an irradiation time of 0.14 s. This approximately corresponds to a temperature profile at a scanning speed of 1 mm s^−1^, for the current focal size of 0.15 mm (Fig. S6). The influence of the laser scanning speed on the temperature profiles of the copper salt layer is simulated and demonstrated in Fig. 4d. The maximum temperature is less than 300 °C at 1 mm s^−1^. The temperature at the center of laser spot further decreased 70 °C, when the scanning speed increased from 1 to 20 mm s^−1^. In all of these conditions, the maximum temperature will not lead the oxidization of copper nanoparticles. Based on these studies, we fixed the laser power at 3 W and the scanning speed at 5 mm s^−1^.

To confirm the thermal decomposition temperature and enthalpy of decomposition of copper nitrate hydroxide, we conducted thermogravimetric analysis in a nitrogen atmosphere, and the curves are illustrated in Fig. 4d. There are two events leading to a total weight loss of 45%. The first one in the interval 60–160 °C, corresponding to an 18% weight loss, is attributed to the removal of weakly bonded water molecules. The second event with the weight loss of 27%, occurring in the 160–350 °C interval, can be assigned to the thermal decomposition and transformation of nitrate ions to nitrogen oxides. In other words, the first step (before 150 °C) is assigned to the loss of water molecules adsorbed in the surface and in the interlayer space. Dehydroxylation and decomposition should take place in the second step, and the temperature is found to be about 245 °C (Fig. 4d) [39]. The TGA results support the experimental and simulation results, which demonstrates that the previous discussion is understandable.

**Fig. 5:**
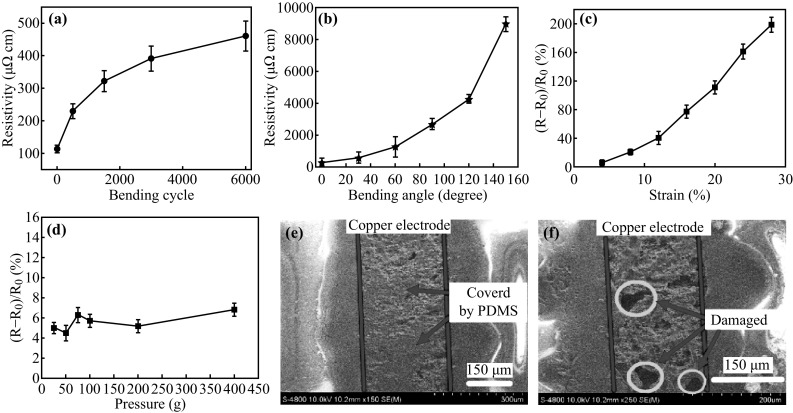
**a** Resistivity as a function of the repeated bending/relaxation cycles. **b** Resistive response of the sensor to the bending angle from 0° to 150°. **c** Resistive response of the sensor to the strain. **d** Response of the sensor to the pressure from 5 to 400 g. **e** SEM images of sandwich structure. The *red lines* describe the copper electrode area, and the *blue arrows* show where the electrode is covered by PDMS. **f** Typical SEM image of copper electrode after 6000 bending cycles. The *blue circles* show the damaged parts of copper electrode. (Color figure online)

After curing the PDMS, the copper pattern is buried into the PDMS surface, indicating a successful transfer of the copper pattern from glass slide to the PDMS elastomer, as well as good adhesion between the copper pattern and PDMS substrate. Figure S7 shows a flexible sensor based on the copper electrode. Owing to the high elasticity of PDMS, the sensor can be easily bended or twisted. The length, width, and thickness of the copper electrode are about 3 mm, 280 μm, and 40 μm, respectively. The resistivity of the Cu patterns was calculated using the following equation: *ρ* = *RS*/*L*, where ρ, *R*, *S*, and *L* are the resistivity, resistance, cross-sectional area, and the length of the copper line. The calculated resistivity was found to be in the range of the copper electrode *ρ* = 87–96 μΩ cm. Although the resistivity is ~40 times higher than that of bulk copper, it is much lower than the values reported for silver nanoparticle electrodes (667 μΩ cm [1]), silver nanowires (~102 μΩ cm [48]), and copper (125 μΩ cm [25]). To observe the stability of copper conductivity, we measured the conductivity in the ambient condition after one month, as shown in Fig. S7c. The resistivity increased to 313 μΩ cm. However, it is still lower than the value achieved for a silver nanoparticle-based sensor [1]. The high conductivity performance after one month can be explained by PDMS protection. By pouring PDMS on copper electrodes, after peeling the sensor from the glass, the cured PDMS still covered the surface of the copper electrode (Fig. 5e), which inhibits oxidation in air [9]. To verify this hypothesis, the resistivities of bare copper electrodes in an ambient environment are measured, and the results are shown in Fig. S8b. It can be observed that the resistivity increased by 30 times, only after two weeks of exposure without the PDMS protection.

The reliability and mechanical robustness of the sensor are shown in Fig. 5. The bendability is measured at a maximal angle of 150° and a deformation frequency of 2 Hz, using reciprocating motion, for 6000 cycles. The real-time resistivity as a function of the bending cycle number is recorded in Fig. 5a. It shows that the electrical resistivity of the sensor increases by more than 4 times to about 440 μΩ cm, after 6000 bending cycles. However, a few holes and microcracks are created after 6000 bending cycles, as shown in Fig. 5e, f. This cracking mechanism is extensively observed in printable electronics [49]. Additional SEM images of the copper electrode on the PDMS are shown in Fig. S9a and b, after bending. When the bending angle is 20°, the surface of the copper electrode does not change, because the elastomer covers the copper surface. Although the microcracks on the surface are not directly investigated, the copper electrode is extruded from the elastomer by the bending force, and the wavy shape of the surface is created at a higher bending angle (Fig. S9b). To study the sensitivity and the elasticity of the sensor, we measured the resistivity under different bending angles, which is illustrated in Fig. 5b. The quantitative sensitivity of the strain sensor can be calculated as *S* = Δ*R*/*R*_0_/*θ*=34 rad^−1^, where *S* and *R*_0_ represent the sensitivity and initial resistance; Δ*R* is the resistance difference before and after bending, and *θ* is the bending angle. Figure 5c illustrates the resistivity curve for the sensor, with a maximum strain of about 28%. The resistance increases by nearly 200 times in the strain range of 0–28%. Furthermore, the gauge factor (GF) is another important parameter that is used to evaluate strain sensor sensitivity. As Fig. 5c shows, the GF is ~8 in the strain range from 0 to 20%. Both S and GF are much higher than the values reported in previous works [9, 21, 48]. In addition, the sensor (1.5 × 1 cm^2^) maintains the same sensitivity at a pressure range up to 400 g, as shown in Fig. 5d. In conclusion, the results of bending, strain, and pressure testing indicate that the nanoparticle-embedded PDMS sensor has strong mechanical robustness.

**Fig. 6:**
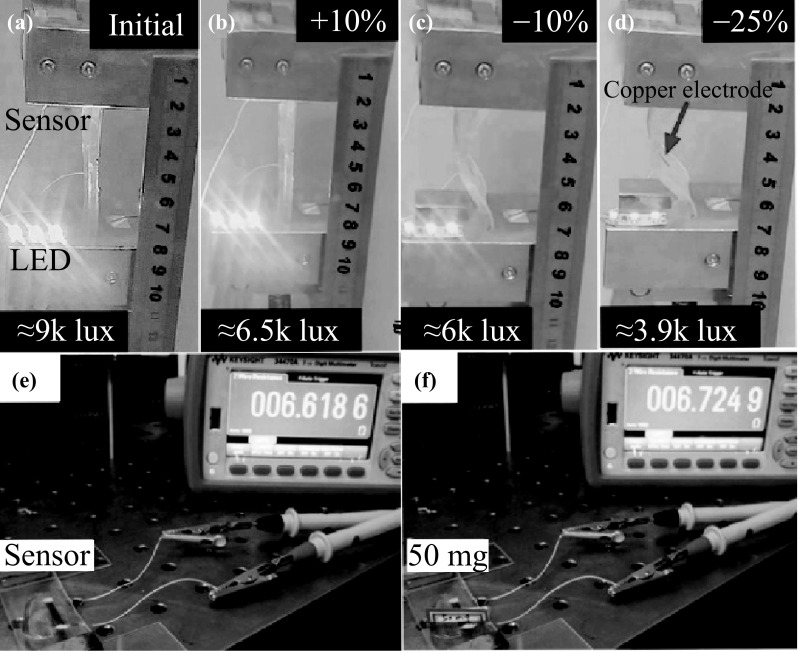
**a**–**d** Illuminance of three LEDs under the stretch of the sensor, from 25 to 10%. **e**–**f** Photographs of resistance response of the sensor by weighting 50 mg paper

These investigations in Fig. 5 verify that the current sensor is much more sensitive than other flexible devices based on networks, when compared with some other works [1, 25, 48]. This characteristic may be attributed to the high sensitivity of the joined copper nanoparticle film to external forces [9, 48, 50]. To verify this high sensitivity, we attached the strain sensor to a tensile tester, and serially connected it with three LEDs, as shown in Fig. 6. When the sensor is bent or stretched, the illuminance of the LEDs decreases rapidly, due to the increase in sensor resistance. In the case of the bending, the light intensity decreased from 9000 to 3900 lx, as the applied strain changed from 0 to 25%. In the case of stretching, it decreases to 6500 lx, at a strain of 10%. Moreover, in order to test the limitation of the ability of the sensor, we left pieces of paper with a total mass of about 50 mg (this value is approximate to a regular mosquito’s mass) on the sensor surface and recorded the resistance using a digital multimeter. The resistance increased by 1.7% due to the weight of the paper on the sensor. It should be noted that this value fulfills the requirement of usual measuring instruments for commercial products.

**Fig. 7:**
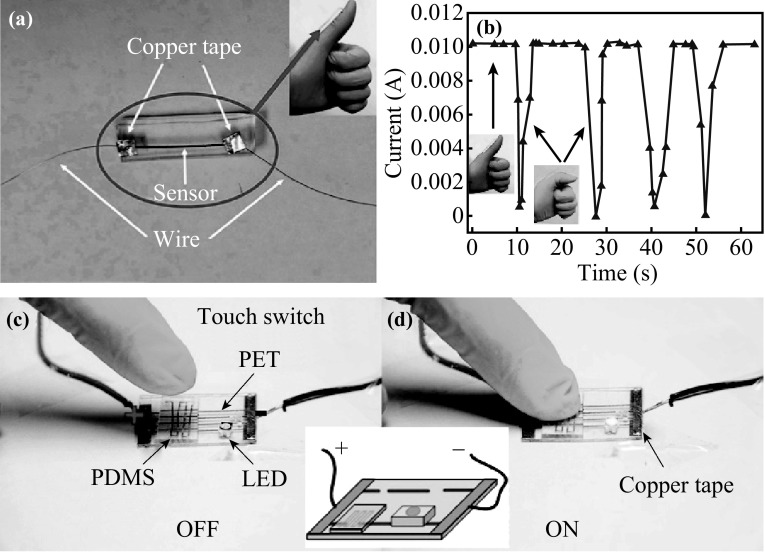
**a** Photograph of the motion detection sensor. **b** Human motion detection of finger with flexible sensor based on copper electrode. **c**–**d** Photograph of touch switch control to turn LED on and off (*Inset* schematic diagram of touch switch)

Finally, we displayed some potential applications of this sensitive sensor, fabricated using our methods. Figure 7a, b shows the real-time detection of thumb motion. The sensor is attached to the glove surface and connected to an electrochemical workstation using copper wire. When the thumb bends, the current is suddenly dropped, due to the increase in resistance. After the thumb straightens again, the current returns to the original value. Different bending angles are thereby deciphered by variable currents. Figure 7c, d shows a pressure-controlled switch, which can turn the LED on and off by pressing the sensor. All the conductive copper lines on the PDMS and PET substrates were fabricated using our developed laser direct writing method. The blue LED was fixed on the copper line using conductive ink. The fabrication process and switching mechanism are illustrated in detail, in Fig. S10. Furthermore, because the reduced copper line is covered by the PDMS with a very thin membrane (Fig. 5e), the laser-reduced copper is electrically insulated from the copper lead wire. In the standby state, the circuit is open, and the LED is off. When touching the sensor surface with a pressure >100 g on PDMS, this thin membrane is deformed, and the copper circuit is electrically connected to the copper lead wire, which results in LED emission.

## Conclusion

In summary, we have developed a new method to fabricate conductive copper electrodes on a PDMS substrate, using one-step laser direct reduction. Owing to the laser heating while scanning, ethylene glycol is decomposed to aldehyde, which reduces the copper (II) to copper (0). A low copper electrode resistivity of about 96 μΩ cm on the PDMS has been achieved, which is comparable to the resistivity based on silver nanowire or carbon nanotube materials fabricated using the ink jetting method. Furthermore, this PDMS-supported copper sensor exhibits excellent performance with ultrahigh sensitivity, under both tensile and compressive strains. The high sensor performance was translated into function device development, in terms of human motion sensing and an electrical switch. The combination of the laser direct reduction of copper ions and the transfer printing on PDMS, for the low-cost production of wearable electronic devices, meets the cost and reliability demands. Therefore, the current study is expected to contribute to the advancing fabrication of flexible electronic devices, for next-generation wearable electronic systems.

## Electronic supplementary material

Below is the link to the electronic supplementary material.

Supplementary material 1 (PDF 1687 kb)
