# Supplementary material for: Laser-Assisted Reduction of Highly Conductive Circuits Based on Copper Nitrate for Flexible Printed Sensors
Source: Nanomicro Lett. 2017 Mar 21;9(4):42. doi: 10.1007/s40820-017-0139-3 (PMC6199039; doi:10.1007/s40820-017-0139-3)
Supplement: Supplementary file 1 — Supplementary material 1 (PDF 1687 kb) [file 40820_2017_139_MOESM1_ESM.pdf]

Supporting Information for

Laser-Assisted Reduction of Highly Conductive Circuits based on Copper Nitrate for Flexible Printed Sensors

Shi Bai[1], Shigang Zhang[2], Weiping Zhou[1], Delong Ma[1], Ying Ma[1], Pooran Joshi[3], Anming Hu[1,4,]*

[1]Institute of Laser Engineering, Beijing University of Technology, 100 Pingle Yuan, Beijing 100124, People's Republic of China

[2]School of Civil and Transportation Engineering, Beijing University of Civil Engineering and Architecture, Zhanlanguan Road, Beijing 100044, People's Republic of China

[3]Oak Ridge National Laboratory, Oak Ridge, Tennessee 37831-6061, USA

[4]Department of Mechanical, Aerospace and Biomedical Engineering, University of Tennessee Knoxville, 1512 Middle Drive, Knoxville, TN 37996, USA

*Corresponding author. E-mail: anminghu@bjut.edu.cn

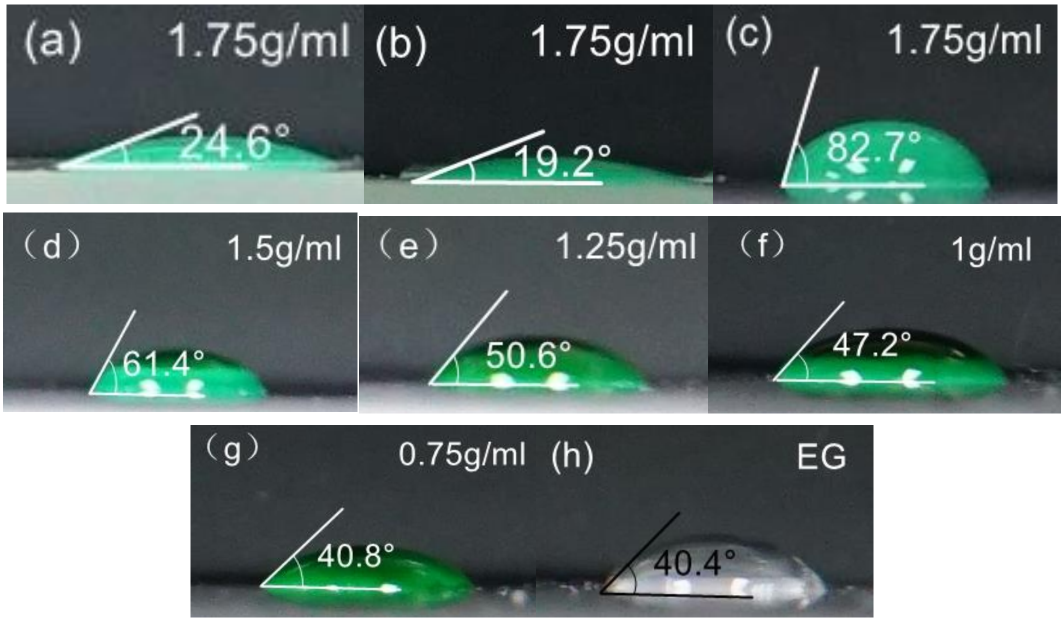

Figure S1 Contact angle of copper salt. The glass substrate **a** treated and **b** did not treated by oxygen plasma. **c-g** Contact angel of different concentration of copper salt on PET substrate. **h** Contact angle of EG on PET substrate

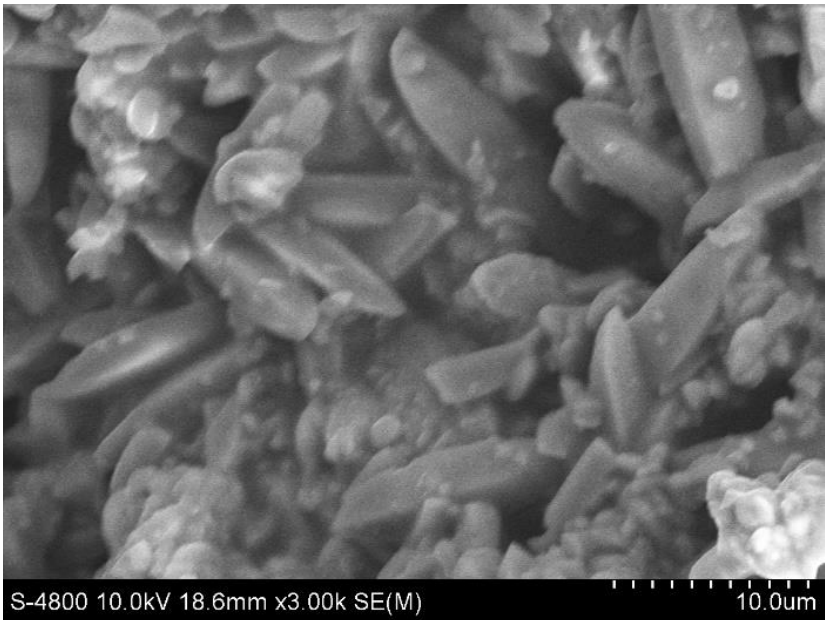

Figure S2 SEM image of copper salt

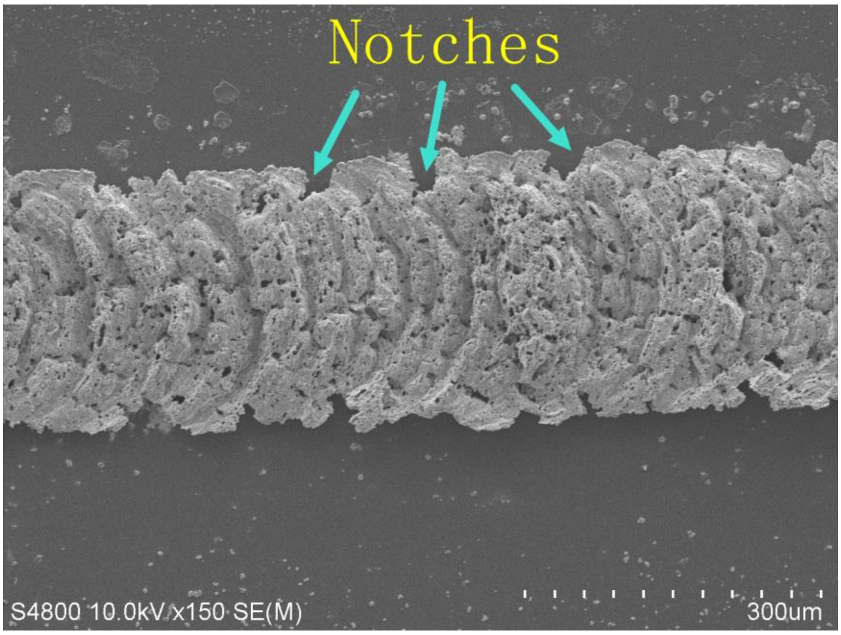

Figure S3 SEM images of copper line on glass reduced by laser at the power of 2 W

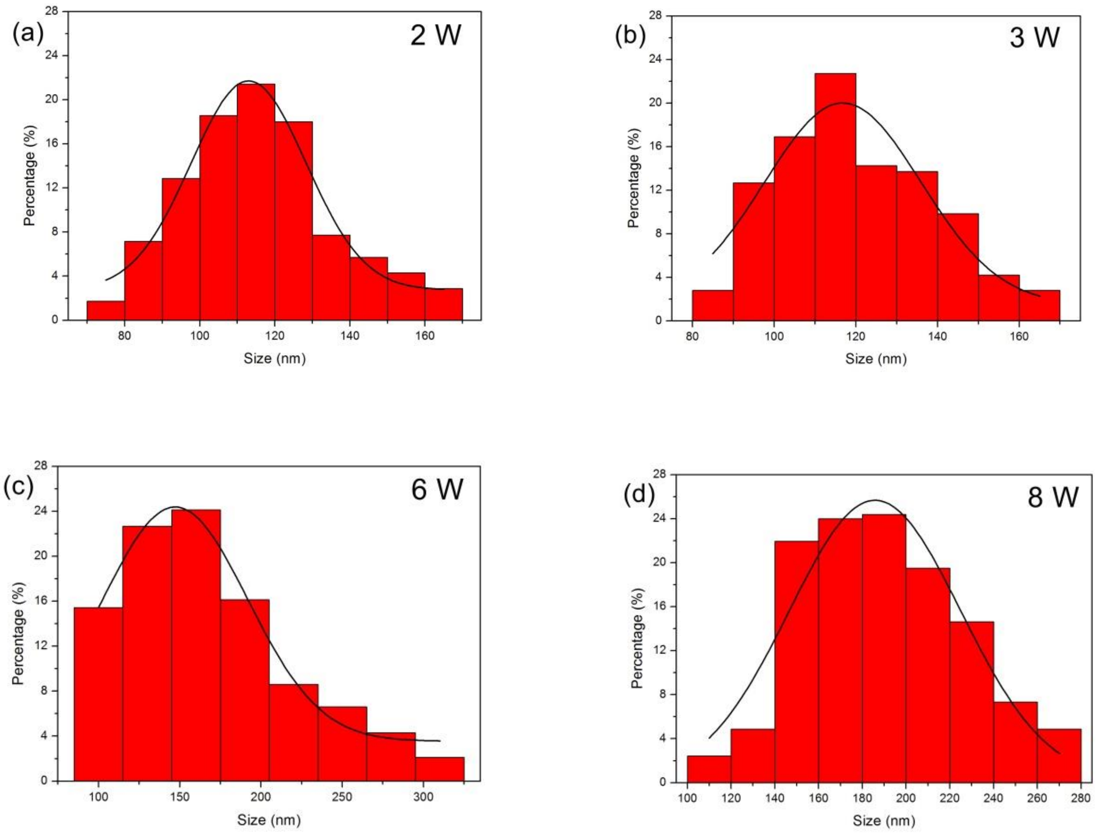

Figure S4 The size distribution of Cu nanoparticles reduced by laser at a power of **a** 2 W, **b** 3W, **c** 6W and **d** 8W

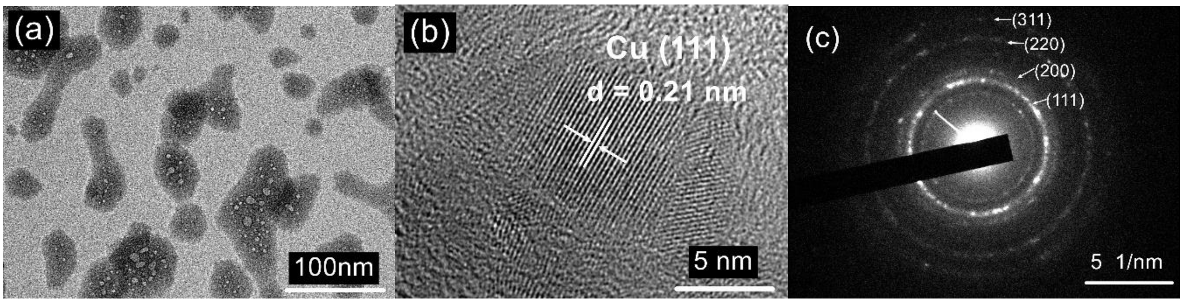

Figure S5 a TEM image of the laser reduced copper nanoparticles. **b** HRTEM image of the {111} copper nanoparticle. **c** SAED pattern of the laser reduced copper nanoparticle

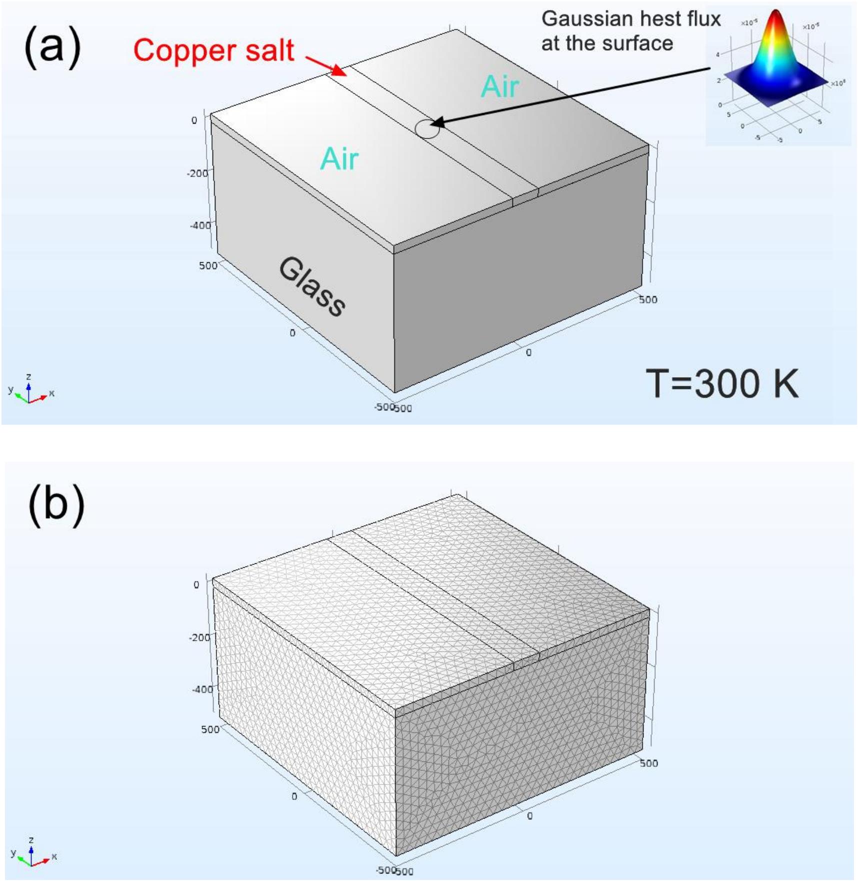

Figure S6 Details on the simulation. **a** Basic simulation parameters. Conjugate heat transfer module with isothermal surroundings (T_0 = 300 K) has been used in every simulation, while the heat source has been fixed as a Gaussian heat source with diameter = 150 μm. **b** Mesh distribution and size of the module

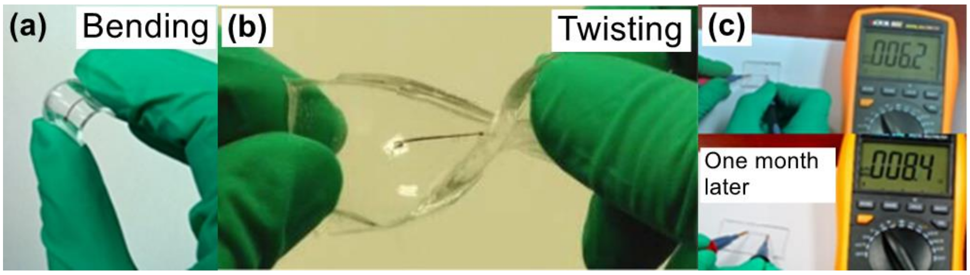

Figure S7 Bending **a** and twisting **b** of a Cu wire sensor. **c** Resistances of the copper electrode measured by a multimeter

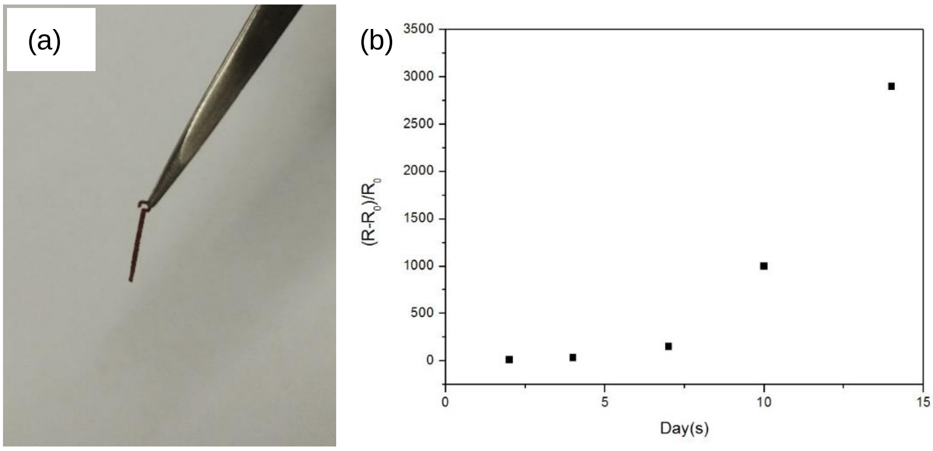

Figure S8 a Reduced copper circuits was picked up by tweezers. **b** The resistivity of bare copper lines changing in the air without any protection

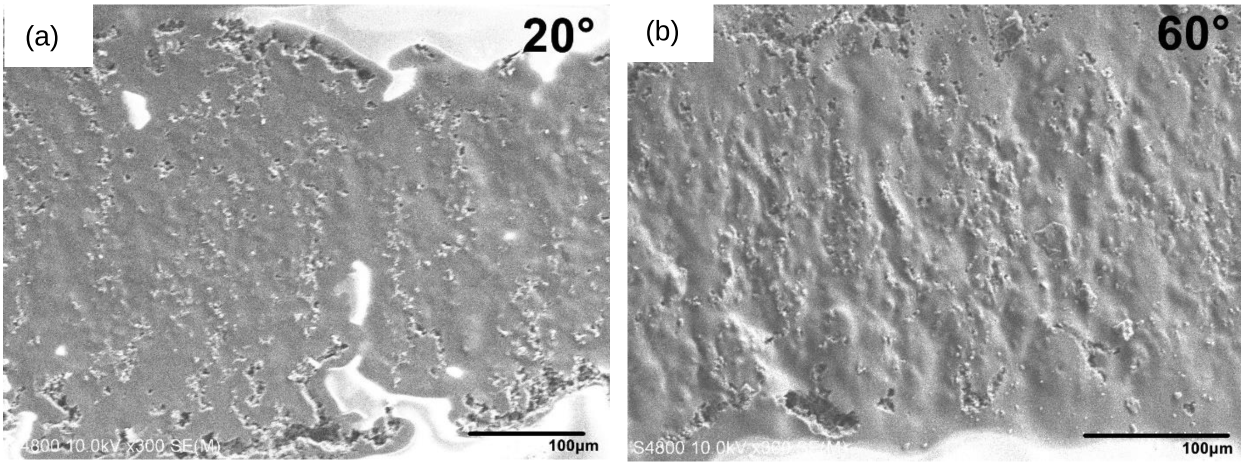

Figure S9 SEM images of copper circuits at a bending angle of **a** 20 °and **b** 60 °

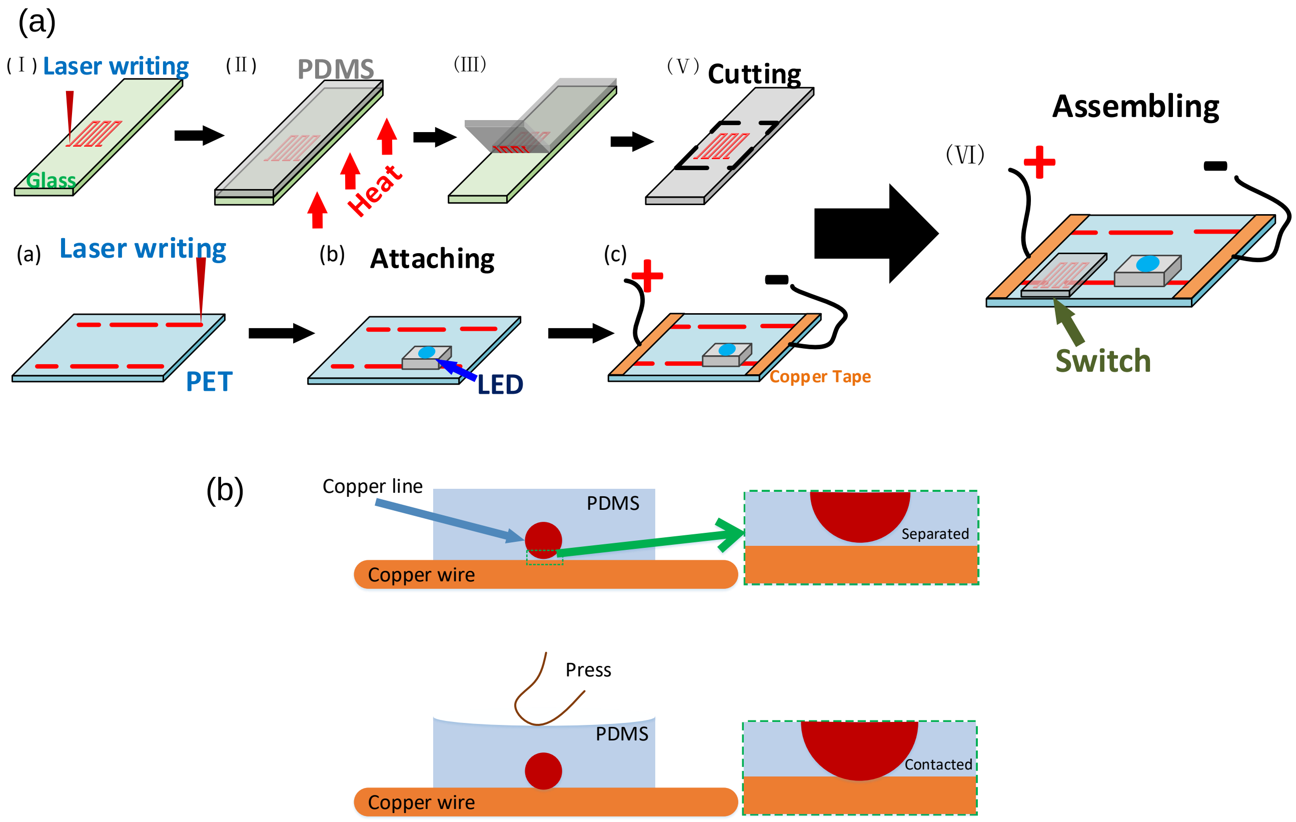

Figure S10 a The fabrication process about flexible device. **b** The operating principle of LED switch based on PDMS and copper electrodes
